# Existe um Papel para Religião e Espiritualidade na Reabilitação Cardíaca?

**DOI:** 10.36660/abc.20230088

**Published:** 2023-03-20

**Authors:** Christina Grüne de Souza e Silva

**Affiliations:** 1 Clínica de Medicina do Exercício Rio de Janeiro RJ Brasil Clínica de Medicina do Exercício (CLINIMEX), Rio de Janeiro, RJ – Brasil

**Keywords:** Doenças Cardiovasculares, Reabilitação Cardíaca, Terapias Espirituais, Qualidade de Vida, Espiritualidade, Religiosidade

A reabilitação cardíaca (RC) é uma intervenção de prevenção secundária para promover a recuperação cardíaca reduzindo a morbidade, mortalidade e incapacidade.^1^ Como tal, a RC foi reconhecida como parte fundamental do cuidado de pacientes com doença cardiovascular e, na maioria das diretrizes atuais de sociedades de cardiologia em todo o mundo, a RC é uma recomendação classe I.^2^

Inicialmente, a abordagem padrão de “reabilitação cardíaca” concentrava-se quase exclusivamente no exercício supervisionado, uma vez que muitos estudos anteriores haviam confirmado um impacto positivo das atividades físicas regulares nos desfechos cardíacos.^3^ No entanto, atualmente, o modelo de RC centrado apenas no exercício tem sido considerado ultrapassado. Como cada vez mais evidências mostram que os eventos cardíacos afetam os pacientes não somente fisicamente, mas também, emocionalmente, socialmente e espiritualmente,^4^os programas contemporâneos de RC são agora vistos como uma intervenção multidisciplinar. Atualmente, abordagens específicas que visam influenciar favoravelmente condições mentais e sociais, aprimorando a qualidade de vida e o bem-estar psicológico, são consideradas peças-chave na recuperação cardíaca em conjunto com componentes centrais bem estabelecidos, como o manejo e controle de fatores de risco cardiovascular, aconselhamento de atividade física e prescrição de exercícios.^5^

A espiritualidade e o envolvimento religioso figuram com destaque entre os métodos que os pacientes cardíacos recorrem ao lidar com o estresse da vida e o adoecimento, estando associados a uma melhor aceitação da doença e a níveis mais baixos de depressão após um evento cardíaco.^6^ Além disso, uma maior religiosidade/espiritualidade tem mostrado ter uma associação protetora com morte relacionada a doenças crônicas, incluindo aquelas causadas por doenças cardiovasculares.^7^ No entanto, a religiosidade raramente é discutida ou avaliada antes ou durante a participação em programas de RC, provavelmente devido à incerteza sobre a sua relevância na prática clínica.

Para preencher essa lacuna, von Flach et al.,^8^ analisaram prospectivamente os dados de uma coorte de 57 pacientes com o objetivo principal de explorar o impacto da espiritualidade e da religião na melhora da aptidão física e da qualidade de vida após participar de um programa de RC de 12 semanas. A espiritualidade e a religião foram avaliadas no início do estudo por meio da aplicação da versão em português (Brasil) do Índice de Religiosidade de Duke, e os valores pré e pós-participação do pico de consumo de oxigênio (VO_2pico_) e do escore *Minnesota Living with Heart Failure Questionnaire (MLHFQ)* foram medidos para avaliar aptidão física e qualidade de vida, respectivamente. Como esperado, após uma mediana de 34 sessões assistidas, observou-se melhora tanto no VO_2pico_ (aumento mediano de 1,6 mL.kg^-1^.min^-1^) quanto na qualidade de vida (redução mediana de 11 pontos no escore do *MLHFQ* ). No entanto, não foram observadas diferenças quanto às principais dimensões da religiosidade ao comparar participantes que atingiram ganhos maiores ou menores em aptidão física ou qualidade de vida. Além disso, não houve correlação entre a mudança no VO_2pico_ e no escore do *MLHFQ* do início ao fim com a religiosidade.

Deve-se ressaltar, no entanto, que esses achados têm capacidade de generalização limitada. Semelhante a pesquisas anteriores sobre religiosidade em diferentes cenários cardíacos,^9 , 10^ o estudo conduzido por von Flach et al.,^8^ teve um pequeno tamanho amostral e uma curta duração de seguimento, além de ter incluído predominantemente homens brancos que participaram de um programa privado de RC em um único centro. Assim, pesquisas futuras são necessárias para entender melhor se, quando e como a religião e a espiritualidade podem desempenhar um papel significativo e independente no manejo de pacientes que participam de programas de RC.
