# Supplementary material for: Existe um Papel para Religião e Espiritualidade na Reabilitação Cardíaca?
Source: Arq Bras Cardiol. 2023 Mar 20;120(3):e20230088. [Article in Portuguese] doi: 10.36660/abc.20230088 (PMC10392855; doi:10.36660/abc.20230088)
Supplement: Translation

# Translation: Is There a Role for Religion and Spirituality in Cardiac Rehabilitation?

**Authors:** Christina Grüne de Souza e Silva

**Affiliations:** 1 Brazil Clínica de Medicina do Exercício (CLINIMEX), Rio de Janeiro, RJ – Brazil

**Keywords:** Cardiovascular Diseases, Cardiac Rehabilitation, Spiritual Therapies, Quality of Life, Spirituality, Religiosity

Cardiac rehabilitation (CR) is a secondary prevention intervention offered to promote cardiac recovery by reducing morbidity, mortality, and disability.^1^As such, CR has been recognized as integral to the comprehensive care of patients with cardiovascular disease, and, in most current guidelines of cardiovascular societies globally, CR is a class I recommendation.^2^

At first, the standard “cardiac rehabilitation” approach focused almost exclusively on supervised exercise, since many previous studies had confirmed a positive impact of regular physical activities on cardiac outcomes.^3^However, currently, the exercise-centric model of CR has been considered outdated. As increasing evidence has shown that cardiac events affect patients not only physically, but also emotionally, socially, and spiritually,^4^contemporary CR programs are now viewed as a multidisciplinary intervention. Among well-recognized core components such as management and control of cardiovascular risk factors, physical activity counseling, and exercise prescription, specific approaches that aim to influence favorably mental and social conditions, improving quality of life and psychological well-being, are now considered key players to cardiac recovery.^5^

Spirituality and religious involvement figure prominently among the methods that cardiac patients call on when coping with life stress and illness, being associated with better disease acceptance and lower levels of disease-related depression after a cardiac event.^6^Moreover, greater religiosity/spirituality has been shown to have a protective relationship with chronic disease-related death, including that caused by cardiovascular disease.^7^Yet, religiousness is rarely discussed or assessed prior to or during participation in CR programs, likely due to uncertainty about its relevance in clinical practice.

To address this gap, von Flach et al.^8^prospectively analyzed data from a cohort of 57 patients with the primary goal of exploring the impact of spirituality and religion on the improvement of physical fitness and quality of life after participating in a 12-week CR program. Spirituality and religion were accessed at baseline through the application of the Brazilian Portuguese version of the Duke Religiosity Index, and pre- and post-participation peak oxygen consumption (VO_2peak_) and Minnesota Living with Heart Failure Questionnaire (MLHFQ) scores were measured to access physical fitness and quality of life, respectively. As expected, after a median of 34 sessions attended, an improvement in both VO_2peak_(median increase of 1.6 mL.kg^-1^.min^-1^) and quality of life (median reduction of 11 points on the MLHFQ score) was observed. However, no differences regarding the major dimensions of religiosity were seen when comparing participants that achieved higher or lower physical fitness or quality of life gains. Moreover, there was no correlation between the change in VO_2peak_and in the MLHFQ score from baseline to end with religiosity.

It should be underscored, however, that these findings have limited generalizability. Similar to previous research regarding religiousness in different cardiac settings,^9 , 10^the study conducted by von Flach et al.^8^had a small sample size and a short follow-up duration, and included predominantly white men that participated in a private single-center CR program. Thus, future research is further needed to understand better if, when, and how religion and spirituality could play a significant and independent role in the management of patients that participate in CR programs.
